# *Ascaris suum* Nutrient Uptake and Metabolic Release, and Modulation of Host Intestinal Nutrient Transport by Excretory-Secretory and Cuticle Antigens In Vitro

**DOI:** 10.3390/pathogens10111419

**Published:** 2021-11-01

**Authors:** Sarina Koehler, Andrea Springer, Nicole Issel, Stefanie Klinger, Michael Wendt, Gerhard Breves, Christina Strube

**Affiliations:** 1 Institute for Parasitology, Centre for Infection Medicine, University of Veterinary Medicine Hannover, 30559 Hannover, Germany; sarina.koehler@tiho-hannover.de (S.K.); andrea.springer@tiho-hannover.de (A.S.); 2 Institute for Physiology and Cell Biology, University of Veterinary Medicine Hannover, 30173 Hannover, Germany; nicole.07@gmx.de (N.I.); stefanie.klinger@gmx.net (S.K.); Gerhard.Breves.iR@tiho-hannover.de (G.B.); 3 Clinic for Swine, Small Ruminants and Forensic Medicine, University of Veterinary Medicine Hannover, 30173 Hannover, Germany; michael.wendt@tiho-hannover.de

**Keywords:** Ascariosis, Ascariasis, soil-transmitted helminths, glucose transport, peptide transport, amino acid transport, malabsorption, excretory-secretory antigen, ES antigen, somatic antigen

## Abstract

*Ascaris suum*, the most important pig parasite, also infects humans as a zoonotic pathogen. Malabsorption upon infection probably results from impaired nutrient transport, presumably mediated by the parasite’s excretory-secretory (ES) or cuticle somatic (CSO) antigens. The present study investigated the electrogenic transport (ΔI_sc_) of glucose, alanine and the dipeptide glycyl-l-glutamine (glygln), as well as glucose net flux rates in pig jejunal tissue after in vitro exposure to adult *A. suum* total ES or CSO antigens in Ussing chambers. ΔI_sc_ of glucose, alanine and glucose net flux rate were significantly decreased after one hour of exposure to total ES antigen. In contrast, CSO antigens increased the transport of glygln. Additionally, nutrient uptake and ES antigen pattern were compared in culture medium from untreated adult worms and those with sealed mouth and anal openings. Untreated worms completely absorbed glucose, while cuticular absorption in sealed worms led to 90% reduction. Amino acid absorption was 30% less effective in sealed worms, and ammonia excretion decreased by 20%. Overall, the results show that *A. suum* total ES antigen rapidly impairs nutrient transport in vitro. Future studies confirming the results in vivo, narrowing down the ES components responsible and investigating underlying molecular mechanisms are needed.

## 1. Introduction

The intestinal roundworm *Ascaris suum* is regarded, economically as the most important porcine parasite, but can also infect humans as a zoonotic pathogen [1]. Economic losses in the pig industry are due to condemnation of livers showing traces of larval migration [2], as well as reduced feed efficiency and weight gain, e.g., [3,4]. Upon oral infection with embryonated eggs, infective third-stage larvae hatch in the gastrointestinal tract and migrate via the liver to the lungs. After tracheal migration larvae reach the small intestine again, where they develop into adult worms [5]. About 6–8 weeks after infection, adult worms start egg-laying and may produce up to 2 million eggs per day, which show a high tenacity in the environment, making ascariosis difficult to control [6,7].

Feed conversion and weight gain may be affected by direct parasite-host competition, e.g., for glucose, which is primarily absorbed via the worm’s cuticle [8]; however, parasite-induced modulation of the hosts’ intestinal nutrient absorption mechanisms may play a more important role. In several studies, a negative impact of intestinal nematode infection on host glucose and protein transport has been shown. For example, the chicken roundworm *Ascaridia galli* inhibited the host´s sodium-coupled intestinal absorption of glucose and alanine as measured in Ussing chamber experiments [9]. Similarly, impaired glucose uptake during murine *Nippostrongylus brasiliensis* infection has been related to a reduced activity of the sodium-dependent glucose transporter SGLT1 [10]. Similar observations were made in another study, which additionally revealed a decreased amino acid and peptide transport in the host´s jejunal epithelial cells [11]. Regarding *A. suum*, infected pigs showed an impaired glucose transport at day 14, 17 and 21 post-infection [12,13].

However, the causal chain leading to impaired nutrient absorption remains largely unexplored, and it is unknown whether changes in the host can be induced rapidly, e.g., upon contact of the mucosa with parasite antigens alone, or whether a prolonged presence of worms, possibly even including larval development, is necessary. To gain insights into these questions, the present study measured nutrient transport in porcine intestinal mucosa after in vitro exposure to *A. suum* excretory-secretory (ES) and cuticle somatic (CSO) antigens. *A. suum* ES antigens have numerous effects on the host, such as modulation of the immune response [14,15,16,17] and inhibition of host blood coagulation during body migration [18]. Therefore, it appears likely that intestinal *A. suum* stages could modulate the uptake of nutrients in the small intestine via ES antigens for their own benefit. Furthermore, nutrient uptake and ES pattern of in vitro cultivated adult worms were investigated. Here, comparison of glucose, amino acid and ammonia culture medium contents from untreated worms with those of worms with sealed mouth and anal openings allowed, among others, insights into nutrient uptake and secretion via the worm’s cuticle.

## 2. Results

### 2.1. Nutrient Uptake and ES Pattern of *In Vitro* Cultivated Worms

**Figure 1:**
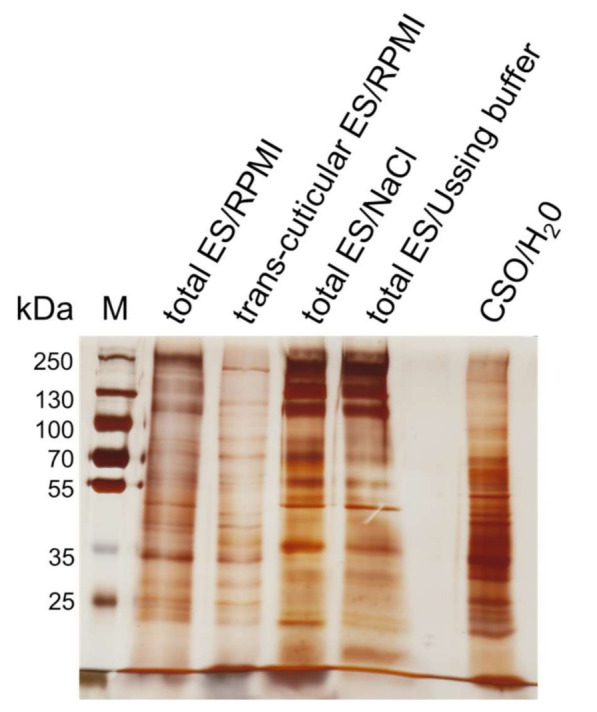
Silver-stained SDS-PAGE loaded with 20 µg ES antigen obtained after worm cultivation in glucose containing RPMI medium, 0.9% NaCl or Ussing buffer and 2 µg CSO antigen per lane. Abbreviations: M, marker (PageRuler™ Plus Prestained Protein Ladder; Thermo Fisher Scientific, Schwerte, Germany); ES, excretory-secretory antigen; CSO, cuticular somatic antigen; H_2_O, bidistilled water.

Total ES, trans-cuticular ES and CSO antigen pattern of in vitro cultivated adult *A. suum* worms visualized by SDS-PAGE and silver staining are shown in Figure 1. Total ES antigens obtained from untreated adult worms after cultivation in 0.9% NaCl or physiological intestinal buffer (Ussing buffer) and total ES antigen obtained by cultivation in RPMI medium and lyophilized to reduce its volume showed comparable banding patterns. A quite similar pattern was also observed for the trans-cuticular ES antigen from RPMI-cultivated worms with sealed mouth and anal openings, representing the ES fractions secreted via the body surface. However, the dominant proportion of high molecular proteins >100 kDa was not observed in the latter.

CSO antigen exhibited a different pattern compared to the ES antigens with the most intense staining in the low and medium molecular weight range (up to 70 kDa).

Nutrient content analyses of RPMI medium obtained after in vitro cultivation of adult untreated *A. suum* showed that the glucose concentration dropped below the measurable range, while a high glucose concentration was observed in native lyophilized RPMI medium (Table 1). After cultivation of worms with sealed mouth and anal openings, a glucose concentration of 28.66 mmol/L was retained in the medium, corresponding to a reduction by more than 91%. Total amino acid content amounted to 90.13 mmol/L in the native RPMI medium, 25.5 mmol/L after cultivation of untreated worms and 61.23 mmol/L after cultivation of sealed worms. Compared to the initial content in the native medium, 16 of 20 amino acids showed decreased concentrations after worm cultivation, with a mean decrease of 46.2% through sealed and 78.0% through untreated worms. Alanine, γ-aminobutyric acid (GABA) and ornithine were not initially present in the RPMI medium but secreted by adult *A. suum*. Interestingly, sealed worms produced more alanine and GABA, while the ornithine content was higher in medium recovered from untreated worms. The content of glutamic acid in the RPMI medium decreased slightly after cultivation of the latter but increased 4.3 times during cultivation of sealed worms. Finally, the ammonia content increased from 48.5 mg/L to 377.2 mg/L after cultivation of sealed worms and to 471.4 mg/L after cultivation of untreated worms, reflecting an increase by about 90% and 87%, respectively.

### 2.2. Functional Measurements upon Exposure of Porcine Jejunal Tissue to Adult A. suum Total ES and CSO Antigens

#### 2.2.1. Impact of Adult *A. suum* Total ES and CSO Antigens on Jejunal Short-Circuit Current (I_sc_/ΔI_sc_) and ^3^H-D-Glucose Net Flux Rates (J_net_)

**Figure 2:**
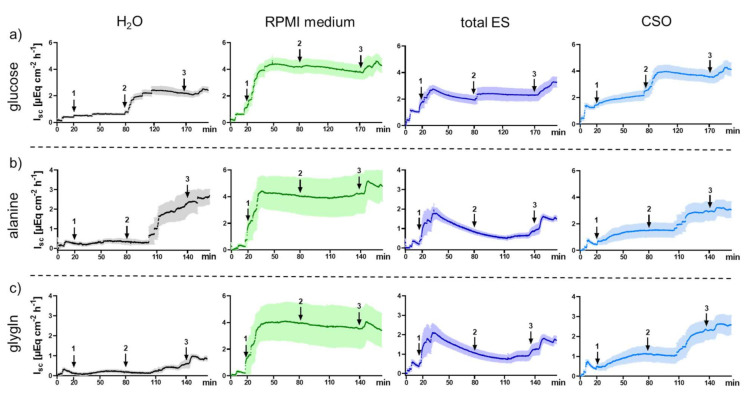
Development of the mean short-circuit currents (I_sc_) of jejunal pig mucosa ± SEM (shaded areas) exposed to adult *A. suum* antigens in the Ussing chamber system. Arrows indicate addition of (**1**) 0.5 mL bidistilled H_2_O (control), native RPMI medium (control), total ES or CSO antigens to the mucosal side; (**2**) 5 mM glucose (**a**), 10 mM alanine (**b**), 10 mM glygln (**c**) to the mucosal side; (**3**) 10 µM forskolin to the serosal side. Abbreviations: ES, excretory-secretory antigen; CSO, cuticular somatic antigen; glygln, glycyl-l-glutamine.

The arithmetic mean values of I_sc_ as a function of time reflecting the electrogenic jejunal transport processes of parasite-naive pigs as measured in Ussing chambers are depicted in Figure 2. After mucosal addition of either bidistilled H_2_O (control), native RPMI medium (concentrated via lyophilization; control), total ES (derived from untreated adult *A. suum* cultivated in RPMI medium and concentrated via lyophilization) and CSO antigen (homogenized cuticles of adult worms in bidistilled H_2_O) 20 min after adjusting the jejunal tissues to short circuit conditions, the initial I_sc_ increase was evaluated during an incubation time of 60 min. The addition of bidistilled H_2_O into the Ussing chambers caused the lowest, barely measurable, I_sc_ increase. In contrast, an addition of RPMI medium caused an initial I_sc_ increase to a high plateau (>4 µEq cm^−2^ h^−1^), probably due to its high glucose content, and therefore did not allow evaluation of the electrogenic response to later addition of the substrates glucose, alanine and the dipeptide glycyl-l-glutamine (glygln). Consequently, the bidistilled H_2_O chambers were used as controls for subsequent statistical analyses regarding the jejunal response to substrate addition in the presence of total ES and CSO antigens. Addition of total ES and CSO antigens into the Ussing chambers also caused an initial I_sc_ increase, but this was less pronounced compared with native RPMI medium. Furthermore, in the total ES antigen chambers, I_sc_ decreased after a marked initial peak.

**Figure 3:**
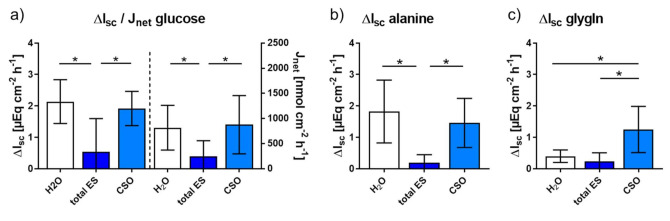
Response of jejunal pig mucosa after 60 min of exposure to bidistilled H_2_O (control) or adult *A. suum* total ES and CSO antigens. Graphs display arithmetic means ± SD of the short-circuit current (ΔI_sc_) in response to glucose (left y-axis) and ^3^H-glucose net flux rate (J_net_; right y-axis) (**a**), ΔI_sc_ in response to alanine (**b**), ΔI_sc_ in response to glygln (**c**). Asterisks indicate statistically significant differences (*p* ≤ 0.05). Abbreviations: ES, excretory-secretory antigen; CSO, cuticular somatic antigen; glygln, glycyl-l-glutamine.

After the addition of glucose, alanine and glygln into the Ussing chambers to quantify the electrogenic response, Δ I_sc_ was calculated by subtracting the basal I_sc_ from the maximal value after substrate addition. In the chambers with bidistilled H_2_O and CSO antigen a similar response was observed, except for a significantly higher Δ I_sc_ in response to glygln after exposure to CSO antigen (*p* = 0.016, Figure 3). In contrast, jejunal exposure to total ES antigen resulted in a significantly lower Δ I_sc_ after glucose (*p* = 0.031 each) and alanine addition (*p* = 0.016 each) compared to both, the bidistilled H_2_O control and the CSO antigen chambers. By contrast, Δ I_sc_ after glygln addition was unaffected by total ES antigen compared to the H_2_O control (*p* = 0.219), but significantly decreased compared to CSO antigen (*p* = 0.016). As mentioned above, the high glucose content of the native RPMI medium and associated transport processes most likely masked or prevented those of the added substrates, so that no electrogenic response could be assessed in these chambers.

Comparison of glucose net flux rates (J_net_) after addition of labelled ^3^H-D-glucose showed that total ES significantly decreased the mucosal-serosal glucose transport compared to H_2_O (*p* = 0.031) and CSO (*p* = 0.016, Figure 3a). In contrast, no significant difference in the glucose net flux rate was observed for CSO in comparison with the H_2_O control (*p* = 0.469).

Tissue viability until the end of the experiments was proven by an I_sc_ increase in all Ussing chamber after the addition of forskolin.

#### 2.2.2. Tissue Conductance (G_t_) in Response to Adult A. suum total ES and CSO Antigens

**Figure 4:**
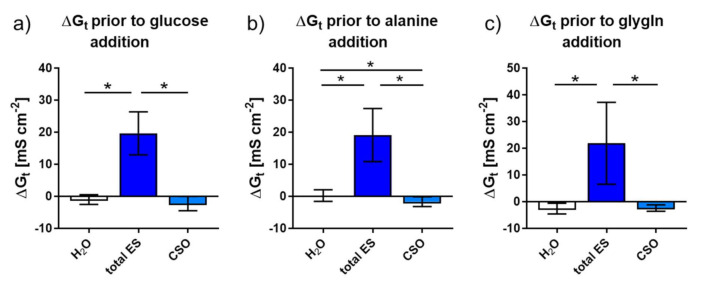
Changes in tissue conductance (ΔG_t_) of jejunal pig mucosa due to exposure to bidistilled H_2_O (control) or adult *A. suum* ES and CSO antigens in Ussing chambers. Results are shown as arithmetic means ± SD and grouped according to subsequent measurements of glucose (**a**), alanine (**b**) or glygln (**c**). Asterisks indicate statistically significant differences (*p* ≤ 0.05). Abbreviations: ES, excretory-secretory antigen; CSO, cuticular somatic antigen; glygln, glycyl-l-glutamine.

Regarding G_t_ as an indicator for tissue permeability, similar changes upon exposure to bidistilled H_2_O (control), total ES and CSO antigens were observed in the different chamber set-ups (Figure 4). Across all set-ups, ΔG_t_ (basal value compared to the maximal G_t_ before the addition of substrates) was significantly higher after the addition of adult *A. suum* ES compared to CSO antigen and H_2_O (*p* = 0.016 each). In Ussing chambers used for alanine transport measurement, CSO antigen exposure led to a significantly decreased G_t_ compared to H_2_O exposure (*p* = 0.031; Figure 4b).

## 3. Discussion

The present study investigated the effect of adult *A. suum* antigens on transport processes in porcine intestinal mucosa by adding them directly into the Ussing chamber system. This approach not only served to identify modulations in nutrient transport upon intestinal roundworm infections in pigs but may also serve as a model for respective human zoonotic infections, and, furthermore, human infections with *A. lumbricoides*. Notably, some authors postulated that *A. suum* and *A. lumbricoides* constitute one species with host adaptations to either pigs or humans, facilitating zoonotic transmission at the animal–human-interface [7,19].

Prior to Ussing chamber experiments, it was verified that the adult *A. suum* total ES antigen used for functional transport assessments was not affected by the lyophilization procedure nor the Ussing buffer. The banding pattern of the lyophilized ES antigen produced in RPMI medium used for the assessments was comparable to non-lyophilized ES antigens produced in physiological saline or mucosal Ussing buffer solution, suggesting that neither the production process nor the Ussing buffer had adverse effects on the functional measurements in the Ussing chamber system. Nevertheless, the experimental ES antigen showed overall weaker bands, which may result from losses during lyophilization or, less probable, reduced production in RPMI medium compared to the other solutions used for worm cultivation.

To gain insights into excretory and secretory processes, more specifically to estimate the contribution of antigens released through the mouth and anal opening of adult worms, total ES antigens were compared to trans-cuticularly ES antigens released by worms with sealed mouth and anal openings. Overall, the banding pattern was quite similar, but often appeared somewhat weaker in the trans-cuticular ES antigen. Especially in the high molecular weight range some bands were barely visible or missing, suggesting that several ES antigen components are (primarily) ex- or secreted via the mouth and anal openings. On the other hand, the sealed worms had lower access to nutrients and may have consequently adapted their metabolism, producing less antigens. Interestingly, the band visible at about 50 kDa in the total ES appeared to be downshifted in the trans-cuticular ES antigen, which might indicate lacking glycosylation or other modifications when released through the cuticle instead of the openings.

Besides ES antigen release, nutrient uptake and release of metabolic products were compared between untreated and sealed worms. Concerning nutrient absorption via the cuticle, amino acid absorption of the sealed worms was reduced by 30% as compared to the untreated ones. However, these numbers are only an approximation, as it remains possible that the tissue glue sealing was incomplete or became loose during cultivation of some specimens. The γ-glutamyl cycle allows the transport of amino acids across membranes via γ-glutamyl transpeptidase activity [20,21]. However, the absorption of proline, glycine and methionine depends on available energy, i.e., glucose, so that a reduced glucose and amino acid absorption are associated with each other [22,23]. Due to the high rate of reproduction, protein synthesis occurs rapidly in *A. suum* and the parasite depends on amino acids to synthesize proteins and as NH2 donors [24]. Worms are capable of anaerobic synthesis of alanine, including the reoxidation of NADH to NAD^+^ by reductive amination from pyruvate, which results from anaerobic glycolysis [25]. Since synthesis of other end products, like urea, requires metabolic energy, some amino acids, including alanine, serve as metabolic end products and fulfil additional functions like pH control, nitrogen excretion, osmotic regulation and intracellular signalling [26]. Interestingly, sealed worms excreted more alanine than untreated worms, which may have been due to lower uptake of glucose. In *A. suum* muscle tissue, starvation increases the activity of glycogen phosphorylase, leading to an increase in pyruvate and thus alanine synthesis [27].

Furthermore, both untreated and sealed worms released GABA, with higher levels in the sealed ones, which also released glutamic acid, while untreated worms did not. In contrast, glutamine levels in the medium decreased in both worm groups. In *A. suum*, glutamine is deaminated to glutamic acid, which is then further converted to GABA [28]. The intensified release of GABA may be beneficial for the nutrient-restricted sealed worms, as it leads to a lower muscle contractility which may be an energy saving strategy [29]. Regarding the high level of glutamic acid release by sealed worms, it may be speculated that glutamic acid served as a collector for excessive amino groups. Normally, it acts as a donor for the formation of ammonia, which usually constitutes about 80% of the total excretory nitrogen aside from urea, and is excreted primarily via the worm´s intestine [30]. Therefore, the reduced ammonia excretion of sealed worms as compared to untreated worms may have been due to the sealed mouth and anal openings on the one hand, and reduced ammonia synthesis on the other hand. Previous research has shown that the relative amounts of ammonia and urea produced by *A. lumbricoides* may vary depending on environmental conditions [31]. Similarly, excretion of the amino acid ornithine, which is formed as part of the urea cycle and can be synthesized from arginine simultaneously with urea [32] was reduced compared to untreated worms.

In the Ussing chamber system, the jejunal nutrient transport was investigated after exposure to total ES and CSO antigens of adult *A. suum* worms, the antigens the jejunal mucosa is mainly confronted with during the patent intestinal phase of roundworm infections.

Glucose transport was assessed by changes in the short-circuit current as well as by direct detection of net flux rates using labelled ^3^H-D-glucose. Initially, lyophilized native RPMI medium was included as a control for ES and CSO antigen exposure. However, the high glucose concentrations in the native medium caused I_sc_ to rise to such a high level that no response to the addition of glucose substrate could be observed in these chambers. Therefore, bidistilled H_2_O was used as a control both for CSO and ES antigens. In contrast, the initial glucose content in the RPMI medium was reduced (close) to zero by the metabolic activity of the cultivated worms, so that no such interference with the Ussing chamber measurements during exposure to total ES antigen could have occurred.

An inhibitory effect of adult *A. suum* total ES antigens on nutrient transport became evident 1 h after exposure. Such inhibitory effects are in line with previous studies on experimental roundworm infections of pigs, which showed decreased glucose absorption, and of chicken, which showed additional alanine transport impairment as well [9,12,13]. Furthermore, tissue conductance was affected. Exposure to total ES antigen significantly increased G_t_, i.e., tissue permeability, while H_2_O as well as CSO antigen decreased G_t_, even into the negative range. An increase in tissue permeability can be expected to increase paracellular nutrient transport, thus, the impairment of nutrient transport due to total ES antigen appears even more striking. The increased G_t_ was probably caused by RPMI medium components in the ES antigen, as exposure to native RPMI medium resulted in a similar G_t_ alteration. *A. suum* total ES antigens impaired both glucose and alanine absorption, similar to results obtained from *A. galli* infected chicken [9]. The impairment of intestinal glucose transport may derive from a change in glucose transporter expression or transporter activity, as shown for *N. brasiliensis* infected mice [10]. Additionally, a reduced intestinal amino acid transporter expression during murine *N. brasiliensis* infection has been found [11]. Due to the short exposure time of only one hour in the current study, it seems less likely that the observed changes resulted from a change in transporter expression. Rather, a change in transporter activity due to phosphorylation of the sodium glucose transporter SGLT1 and the amino acid transporter ASCT1 by protein kinase A may explain the observed changes [33,34,35]. Unfortunately, no Ussing chamber experiments could be performed with trans-cuticular ES antigen because of the limited number of chambers and corresponding devices available. Likewise, after testing total ES and CSO antigens against H_2_O and RPMI as (intended) controls, no further experiments could be performed, since the tissues mounted into the chambers must be absolutely fresh. Thus, we were restricted to total ES and CSO effects on nutrient transport.

In contrast to total ES antigen, CSO antigen did not significantly affect glucose nor alanine transport. However, exposure to CSO antigen increased transport of the dipeptide glygln across the jejunal mucosa, which constitutes a new finding concerning somatic antigens of nematodes. It may be speculated that the phosphorylation status and thus activity of the Na^+^/H^+^-exchanger 3 (NHE3) was affected, subsequently altering the intracellular pH and the function of peptide transporter 1 (PepT1) [36,37]. Taken together, the results of this study support the hypothesis that *A. suum* ES antigens have a stronger impact on host cells than somatic antigens, similar to findings regarding their immunogenicity [14,15].

## 4. Materials and Methods

### 4.1. Production of A. suum Total ES, Trans-Cuticular ES and CSO Antigens

Adult *A. suum* specimens were obtained from the intestine of pigs slaughtered at an abattoir. To obtain total ES antigen, 210 worms (male-female ratio, 1:2) were cultivated in groups of five in cell culture flasks containing RPMI 1640 medium supplemented with 300 mg/L L-glutamin, 2000 mg/L sodium bicarbonate and 2000 mg/L glucose (PAN Biotech, Aidenbach, Germany) at 37 °C and 5% CO2 for 72 h. Every 24 h, the culture medium was collected and replaced by fresh medium. Collected medium was centrifuged (2750× *g* for 20 min) and the supernatant was sterile-filtered through low-protein binding disposable sterile bottle top filters with a descending pore size of 0.6, 0.45 and 0.2 µm (Sarstedt, Nümbrecht, Germany). The filtrate was pooled and frozen at −80 °C in aliquots of 1000 mL before concentration via lyophilization (Alpha 1-2 LDplus, Martin Christ Gefriertrocknungsanlagen, Osterode, Germany). Each aliquot was reduced to 50 mL during the lyophilization process. The concentrated medium was stored at −80 °C until further use. Additionally, 1000 mL native RPMI medium, i.e., cultivation medium without worms, was lyophilized to 50 mL to serve as a control in further analyses.

To investigate transport processes via the parasite’s cuticle, in the same number of adult worms the mouth and anal openings were sealed with surgical glue (Histoacryl, B. Braun, Tuttlingen, Germany). These worms were cultivated as described but kept in individual cell culture flasks to prevent loosening of the glue plugs due to frictions between moving parasites. Recovered medium containing trans-cuticular ES antigen was sterile-filtered and lyophilized as described above.

For comparative purposes, *A. suum* total ES antigens were also produced in nutrient-free 0.9% NaCl solution as well as physiological intestinal buffer pH 6.4 (for ingredients see section on Ussing chamber measurements below). For this purpose, 90 adult worms each (male-female ratio, 1:2) were kept in 400 mL 0.9% NaCl or 500 mL physiological intestinal buffer, respectively, for 24 h at 37 °C and 5% CO_2_. Afterwards, the supernatants were stored at −80 °C fur further use, but no lyophilization was performed.

CSO antigen was recovered from the cuticles of 210 adult worms (male-female ratio, 1:2). First, the cuticle was stripped off the worms, then rapidly frozen in liquid nitrogen and homogenized using a mortar and pestle. The obtained powder was suspended in 700 mL bidistilled H_2_O.

### 4.2. Nutrient Uptake and ES Pattern of *In Vitro* Cultivated Adult Worms

The obtained antigen fractions were separated by SDS-PAGE using 20 µg of total or trans-cuticular ES and 2 µg CSO per lane and visualized by silver staining. To determine protein contents, Bradford Assay (Pierce™ Detergent Compatible Bradford Assay, Thermo Fisher Scientific, Waltham, MA, USA) and spectrophotometric quantification (NanoDrop™ 1000 spectrophotometer, PEQLAB Biotechnologie, Erlangen, Germany) were performed.

As the RPMI medium initially contained 2000 mg/L glucose, the residual glucose content after worm cultivation was measured (Glucose Colorimetric Detection Kit, Thermo Fisher Scientific, Schwerte, Germany) to determine glucose absorption during in vitro cultivation and to check for possible interference with Ussing chamber measurements. Furthermore, amino acid and ammonia contents of lyophilized native RPMI medium as well as lyophilized medium after worm cultivation were analysed by custom service (Gesellschaft für Lebensmittel-Forschung, Berlin, Germany).

### 4.3. Animals and Sample Collection

Seven German landrace hybrid pigs (10 weeks of age, approximately 30 kg body weight, 4 males and 3 females) were obtained from the Ruthe Research and Education Farm of the University of Veterinary Medicine Hannover, which is free of *Ascaris suum*. Pigs were housed in a group and received a standard pig diet (Deuka Ferkelstarter Primo, Düsseldorf, Germany) ad libitum until necropsy. To verify that the animals were helminth-naive, faecal samples were collected at the day of arrival and necropsy (day 9–29) and examined with the combined sedimentation-flotation method [38]. Additionally, serum was collected during necropsy for serological analysis. As no serodiagnostic test for pigs was available on the market at that time, a human test was carried out (Human Anti-*Ascaris lumbricoides* IgG ELISA, Abcam, Amsterdam, the Netherlands). Such test was considered suitable due to immunological cross-reaction between *A. lumbricoides* antigen and *A. suum* antibodies [19,39]. No parasite eggs were found in the coproscopical analyses, and all pigs tested seronegative.

Pigs were euthanized by bolt shot and subsequent exsanguination. The intestines were immediately removed and a 120 cm long segment, approximately at the sixth meter of the jejunum, was collected, rinsed with ice-cold 0.9% NaCl and stored in serosal buffer (see section below) for subsequent use in Ussing chamber experiments.

### 4.4. Ussing Chamber Measurements

The jejunal segments were opened at the mesenterial side, stripped of the serosa and muscle layer, and prepared sections were mounted into Ussing chambers with an exposed area of 1.00 cm^2^ [40]. On the serosal side, the buffer solution (pH 7.4, 290 mosm/l) contained (mM): 113.6 NaCl, 5.4 KCl, 0.2 HCl, 1.2 MgCl_2_, 1.2 CaCl_2_, 21 NaHCO_3_, 1.5 Na_2_HPO_4_, 1.2 mannitol, 7.0 4-(2-hydroxyethyl)-1-piperazineethanesulfonic acid (HEPES), 5.0 glucose, 6.0 Na-gluconate. For glucose measurements, the buffer on the mucosal side (292 mosm/l, pH 7.4) contained (mM): 113.6 NaCl, 5.4 KCl, 0.2 HCl, 1.2 MgCl_2_, 1.2 CaCl_2_, 21 NaHCO_3_, 1.5 Na_2_HPO_4_, 1.2 mannitol, 20 HEPES. For measurement of amino acid and dipeptide transport, the mucosal buffer (300 mosm/l, pH of 6.4) contained (mM): 113.6 NaCl, 5.4 KCl, 0.2 HCl, 1.2 MgCl_2_, 1.2 CaCl_2_, 2 NaHCO_3_, 0.37 Na_2_HPO_4_, 1.13 Na_2_HPO_4_, 1.2 mannitol, 19.83 Na-gluconate, 32.94 mannitol.

Tissues were aerated with carbogen (95% CO_2_, 5% O_2_) and temperature was held at 37 °C during the entire experiments. A computer-controlled voltage clamp device (Mussler Scientific Instruments, Aachen, Germany) was used to measure short-circuit current (I_sc_) and tissue conductance (G_t_). After mounting the chambers, 5–10 min of equilibration was allowed before potential differences were set to 0 mV. After 20 min of equilibration under voltage-clamped conditions, either 500 µL bidistilled H_2_O (control), lyophilized native RPMI medium (control), or ES antigens or CSO were added to the mucosal side of selected chambers and incubated for one hour. Twelve chambers per pig were used for measurements of glucose transport, in the following set-up: four chambers supplemented with total ES antigen, four chambers with CSO antigen, two chambers with bidistilled H_2_O (controls) and two chambers with lyophilized native RPMI medium (controls). For transport measurements of alanine and glycyl-L-glutamine (glygln), respectively, six chambers per pig were applied as follows: two chambers with total ES antigen, two chambers with CSO antigens, one chamber with bidistilled H_2_O (control) and one chamber with lyophilized native RPMI medium (control).

For assessment of glucose transport, 5 mM glucose was added one hour after exposure to the antigens or controls to the mucosal side. This added amount of glucose did not induce a relevant proportion of paracellular glucose transport at the epithelium [41]. To prevent osmotic effects, 5 mM mannitol was simultaneously added to the serosal side. ΔI_sc_ and ΔG_t_ were calculated by subtracting the basal value from the maximal value after substrate addition.

In addition, unidirectional glucose flux rates were determined by adding 5 µCi ^3^H-D-glucose (185 kBq; Perkin Elmer, Rodgau, Germany) either to the mucosal or the serosal side 10 min after addition of the 5 mM unlabelled glucose. Immediately after addition as well as 60 min later, a 50 µL-sample was taken from the radioactive side and mixed with 450 μL unlabelled buffer, while four samples of 500 µL each were taken in 15 min intervals from the unlabelled side. The removed volume was replaced with glucose-containing buffer. Samples were mixed with 4.3 mL scintillation liquid (Rotiszint^®^ eco plus LSC-Universalcocktail, Carl Roth, Karlsruhe, Germany) and radioactivity was measured in decays per minute (dpm) (Tri-Carb 2500 TR Liquid Scintillation Analyser, Packard Instrument Company, Downers Grove, USA). Unidirectional flux rates were calculated by using standard equations [42], and the serosal-to-mucosal flux rate (J_sm_) was subtracted from the mucosal-to-serosal flux (J_ms_) to obtain the net flux rate (J_net_).

For the assessment of amino acid transport, the glucose protocol was applied, but 100 µL alanine (10 mM) was added instead of glucose to the mucosal side. Peptide transport was assessed by adding 200 µL glygln (10 mM, mucosal side) after 30 min of preincubation (directly after 60 min exposure period to respective antigen or control) with amastatin (3-amino-2-hydroxy-5-methylhexanoyl-L-valyl-L-valyl-L-aspartic acid, 10 μM, mucosal side), which inhibits dipeptide cleavage [43]. To balance the osmolality, the respective amount of mannitol was added to the serosal side. Again, ΔI_sc_ was calculated by subtracting the basal value before substrate addition from the maximal value after addition. At the end of each experiment, forskolin (10 μM, serosal side, Sigma-Aldrich, Taufkirchen, Germany) was added to assess tissue viability.

### 4.5. Statistical Analyses

Arithmetic means ± SD of short-circuit current (ΔI_sc_) and glucose net flux rates (J_net_) were calculated. Comparisons between antigen-exposed and control mucosal segments were performed by Wilcoxon rank sum test for paired data using GraphPad Prism (v. 8.0.1, San Diego, CA, USA). Differences were regarded as statistically significant if *p* < 0.05.

## 5. Conclusions

This study investigated impairment of host nutrient absorption by *A. suum* antigens and nutrient uptake by the parasite. The results allow us to gain further insight into the causal chains leading to reduced nutrient absorption during roundworm infection. For the first time, an immediate effect of *A. suum* antigens on the transport processes of the jejunal mucosa has been shown. The decreased glucose and alanine transport across the epithelium after exposure to total ES antigen in Ussing chambers confirmed previous studies on roundworm infected pigs and chicken, as well as nematode infected rodents. An unexpected finding was the increased transport of the dipeptide glygln after exposure to the CSO antigen. Given the short exposure period of only one hour, the observed changes were probably mediated by alterations in the activity of glucose, alanine or peptide transporters, rather than by changes in transporter expression. Further studies are needed to confirm the effect of *A. suum* antigens on transport processes in vivo, to narrow down the ES components responsible for the observed changes, and to determine underlying molecular mechanisms. Comparative analysis of ES products and culture media from untreated worms and such with sealed mouth and anal openings indicated that a certain ES fraction is secreted via the cuticle, and that nutrient restriction affects the metabolic processes of *A. suum*.

## Figures and Tables

**Table 1:** Glucose, amino acid and ammonia content of lyophilized native RPMI medium and lyophilized RPMI medium after in vitro cultivation of adult *A. suum* with untreated (containing total ES antigen products) and sealed mouth and anal openings (containing trans-cuticular antigen products only).

Analyte	Native Lyophilized RPMI (mmol/L)	After Cultivation of Untreated Worms (mmol/L)	% Difference	After Cultivation of Sealed Worms (mmol/L)	% Difference
Glucose	325.82	<0.01	>99.9	28.66	91.2
Alanine	<0.01	**1.22**	**>99.2**	**3.17**	**>99.7**
γ-aminobutyric acid (GABA)	<0.01	**0.18**	**>94.4**	**1.42**	**>99.3**
Arginine	20.44	6.22	69.6	16.26	20.5
Asparagine	5.68	<0.01	>99.8	0.06	98.9
Aspartic acid	2.23	<0.01	>99.6	0.56	74.9
Glutamic acid	1.81	1.62	10.5	**7.77**	**76.7**
Glutamine	29.26	4.94	83.1	14.14	51.7
Glycine	1.92	0.32	83.3	0.79	58.9
Histidine	1.21	0.54	55.4	0.91	24.8
Isoleucine	5.44	2.42	55.5	4.10	24.6
Leucine	5.38	1.88	65.1	3.76	30.1
Lysine	2.96	0.15	94.9	0.93	68.6
Methionine	1.16	0.23	80.2	0.66	43.1
Ornithine	<0.01	**1.37**	**>99.3**	**0.40**	**>97.5**
Phenylalanine	1.17	0.34	70.9	0.64	45.3
Proline	1.97	0.92	53.3	1.76	10.7
Serine	3.92	<0.01	>99.7	0.05	98.7
Threonine	2.31	<0.01	>99.6	0.39	83.1
Tyrosine	1.40	0.36	74.3	0.91	35.0
Valine	1.87	1.08	42.2	1.58	15.5
Ammonia	48.5 mg/L	**471.4 mg/L**	**89.7**	**377.2 mg/L**	**87.1**

Analytes obviously secreted by the worms are shown in bold for easier differentiation.

## Data Availability

Data supporting reported results is contained within the article.
